# Dynamic stabilization in adolescent idiopathic scoliosis with a 5-year follow-up: a case report

**DOI:** 10.1186/s13256-025-05326-8

**Published:** 2025-07-01

**Authors:** Lei Luo, Liehua Liu, Pei Li, Chen Zhao, Lichuan Liang, Yongjian Gao, Qiang Zhou

**Affiliations:** Department of Orthopedics, The Third Affiliated Hospital of Chongqing Medical University, Chongqing, China

**Keywords:** Dynamic stabilization, Dynesys, Adolescent idiopathic scoliosis, Lenke 5, Case reports

## Abstract

**Background:**

Although instrumented fusion is the most widely accepted surgical treatment for adolescent idiopathic scoliosis, it leads to permanent spinal motion loss and an increased risk of adjacent segment degeneration. Consequently, there is great interest in finding nonfusion methods to correct scoliosis in patients with adolescent idiopathic scoliosis. The aim of this manuscript is to report a case of adolescent idiopathic scoliosis (Lenke 5C) treated by dynamic stabilization without fusion using the Dynesys system.

**Case presentation:**

The patient was a 17-year-old East Asian female. Before the operation, the major lumbar curve was 32.2° and the lumbosacral curve was 18.5°, with the Risser sign at grade 4. The procedure was performed using the Wiltse approach. The extent of fixation was from the cephalic horizontal vertebra to sacrum. The scoliosis was corrected by the unequal length of the spacers on the concave/convex side. We obtained a good correction of scoliosis, which was maintained during the 5-year follow-up. We also observed less intraoperative blood loss, faster postoperative recovery, and more motion preservation.

**Conclusion:**

In this case, dynamic stabilization was demonstrated to be technically feasible for the treatment of thoracolumbar/lumbar moderate scoliosis. The benefits are related to less damage to the soft tissues, reduced blood loss, and motion preservation. However, further studies are needed to determine the effectiveness of the described surgical strategy.

## Background

Current conventional treatments for adolescent idiopathic scoliosis (AIS) include observation, racing, or spinal fusion [1]. Although spinal fusion is a relatively safe and effective procedure with the capability to achieve and maintain substantial three-dimensional correction, it results in permanent loss of spinal motion [2] and may impede spinal growth [3, 4]. There is also a concern for accelerated disc degeneration in uninstrumented segments [5]. As such, there is great interest in finding definitive nonfusion methods to correct scoliosis in patients with AIS. The nonfusion surgical methods reported in previous literature include anterior vertebral body tethering (AVBT) [6], vertical expandable prosthetic titanium ribs (VEPTR) [7], and growing-rod [8]. However, they are only applicable to early onset scoliosis and skeletally immature patients. 

**Fig. 1:**
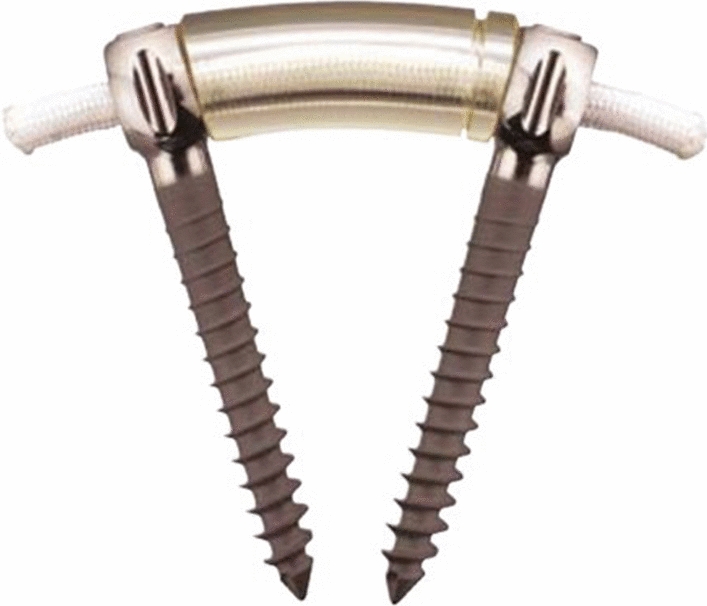
The Dynesys system consists of titanium alloy screws, polyethylene terephthalate cords, and hollow cylinder polycarbonate urethane spacers

In recent years, a dynamic stabilization system (Dynesys system, Fig. 1) has been introduced to overcome the drawbacks of fusion in the treatment of degenerative lumbar scoliosis. Several studies have shown that dynamic stabilization could correct the scoliosis and prevent the progression of the curve while preserving some mobility [9–12]. However, there has been limited literature reporting on the application of dynamic stabilization in the treatment of adolescent idiopathic scoliosis. Surgeons are primarily concerned about the ability to correct deformities with the Dynesys system as well as the risk of screw loosening and breakage in the long term. The aim of this manuscript is to present the treatment results for a patient with AIS treated by dynamic stabilization without fusion.

### Case presentation

**Fig. 2:**
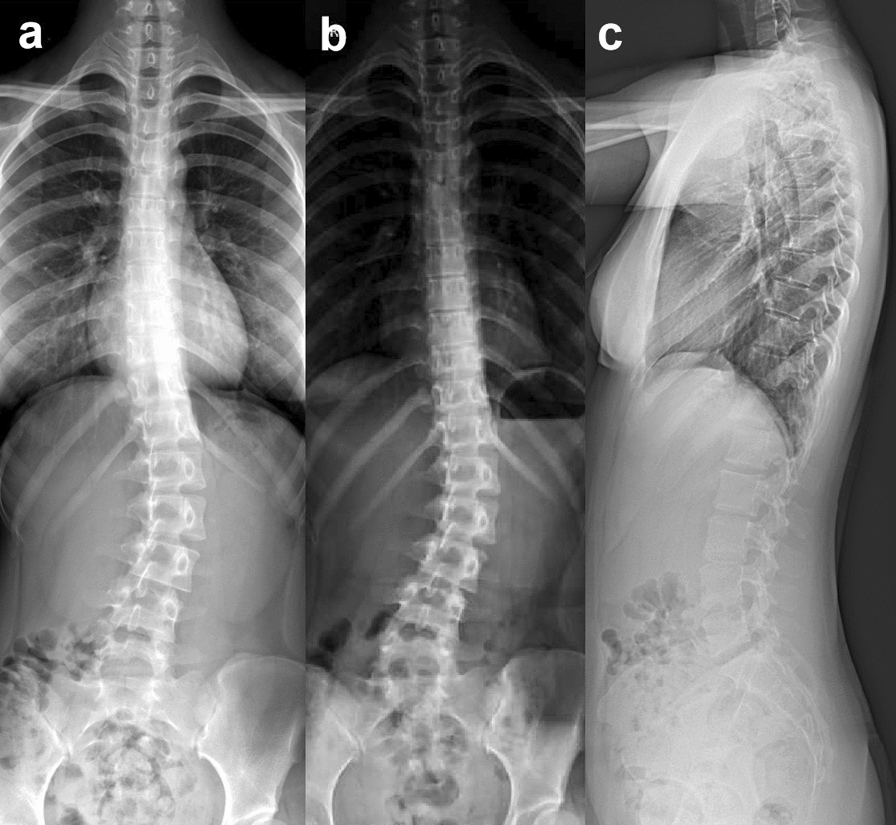
X-ray of the entire spine of a 17-year-old female showed Lenke 5C idiopathic scoliosis; **a** 1 year pre-admission; **b**, **c** on admission

**Fig. 3:**
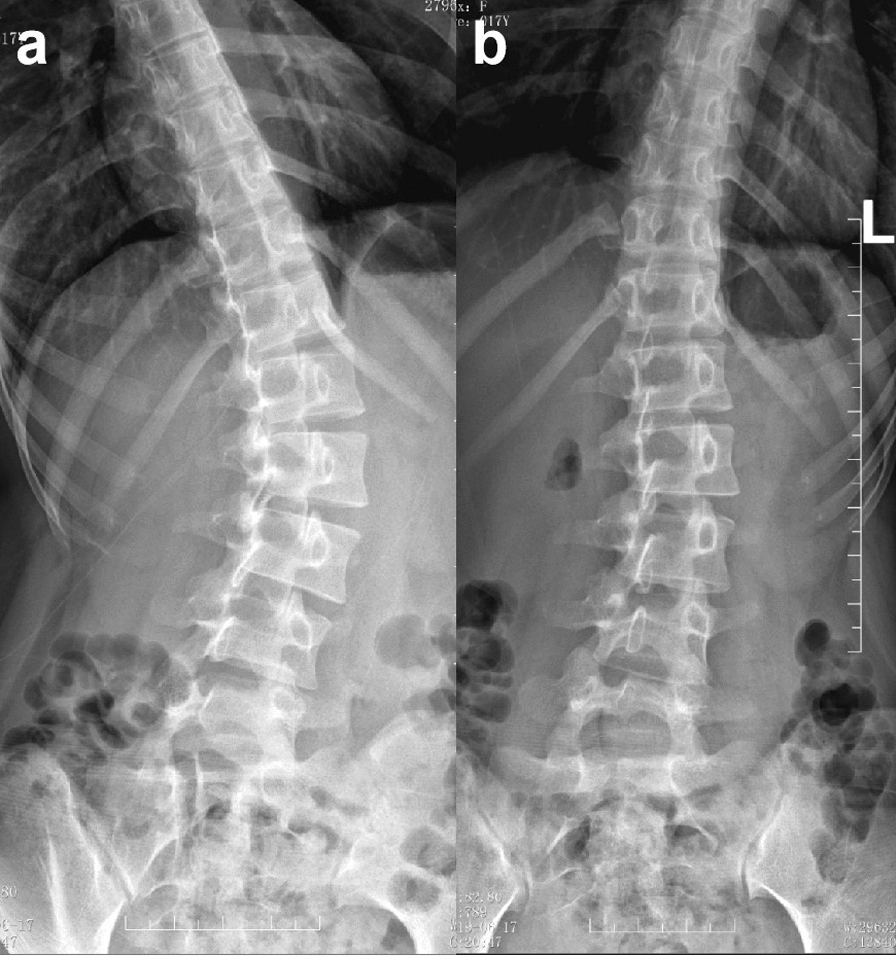
The right lateral bending X-ray showed that the lumbosacral curvature was 15.1° (**a**), and the left lateral bending X-ray showed that the lumbar curvature was 4.6° (**b**). The flexibility of the main lumbar curvature was 85.7%

The patient was a 17-year-old East Asian female, with a height of 162 cm and a weight of 51 kg. She had no relevant diseases, and there was no history of scoliosis in her family. She was diagnosed with AIS Lenke type 5C, with the major lumbar curve measuring 27.6° (Fig. 2a), 1 year prior to admission. The doctor recommended observation, chin-ups, and core muscle strength training, but no improvement was perceived. Therefore, the patient and her parents visited the doctor again and sought other treatment methods. On admission, the patient had recurring episodes of low back pain without neurological symptoms. She presented with asymmetry in the bilateral back and a prominence on the left side when bending forward (Fig. 7a). Therefore, the patient suffered from psychological stress due to the deformity. The anteroposterior X-ray of the entire spine showed the major lumbar curve was 32.2°, and the lumbosacral curve was 18.5° (Fig. 2b). On the alleviated bending X-ray, the lumbar curve was 15.1°, and the lumbosacral curve measured 4.6° (Fig. 3). The Nash–Moe rotation classification of the apical vertebra was grade II, while the Risser sign was grade 4. The lumbar lordosis angle was 41.6°.

Prior to the surgery, informed consent was obtained from the patient and her parents, and ethical approval was granted by the Hospital’s Ethics Committee. The patient underwent surgery on 19 June 2019. During the procedure, the patient was placed in the prone position under general anesthesia. We performed surgical correction through the Wiltse approach with posterior median skin incision. After incising the skin and subcutaneous tissue, an incision was made in the lumbar dorsal fascia approximately 3 cm lateral to the spinous process. Subsequently, the multifidus muscle and the longissimus muscle were bluntly dissected to expose the screw entry point of pedicle screws. The entry point was located at the junction of the lateral border of the superior articular process and the base of the transverse process. The range of internal fixation ranged from the cephalic horizontal vertebrae to sacrum. Then the patient’s position was modified to obtain the appropriate lumbar lordosis. The polycarbonate urethane spacers were cut according to the measured distance between the pedicle screws (longer than measured on the concave side and shorter on the convex side). The spacers were then inserted together with the polyester cords between the screws. Finally the system was tightened under correct compression. Two drainage tubes were placed inside the incision. Then the wound was copiously irrigated before being closed in layers. The duration of the surgery was 192 minutes, and the intraoperative blood loss was 200 mL. The patient was allowed to get up 2 days after surgery when the drainage tubes were removed. She was discharged 6 days after surgery, whereas a stiffer waist support was prescribed for 3 weeks. 

**Fig. 4:**
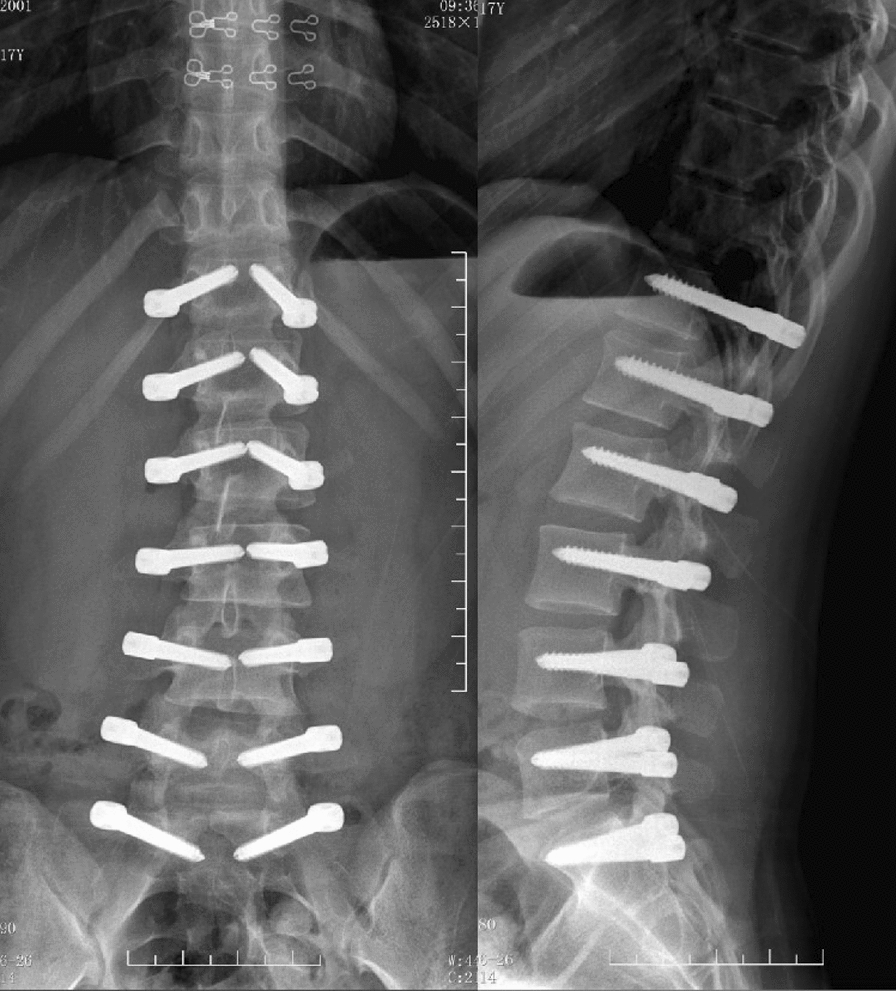
After surgery, the X-ray showed that the lumbar curvature was 3.6°, and the lumbosacral curvature was 0.4°

**Fig. 5:**
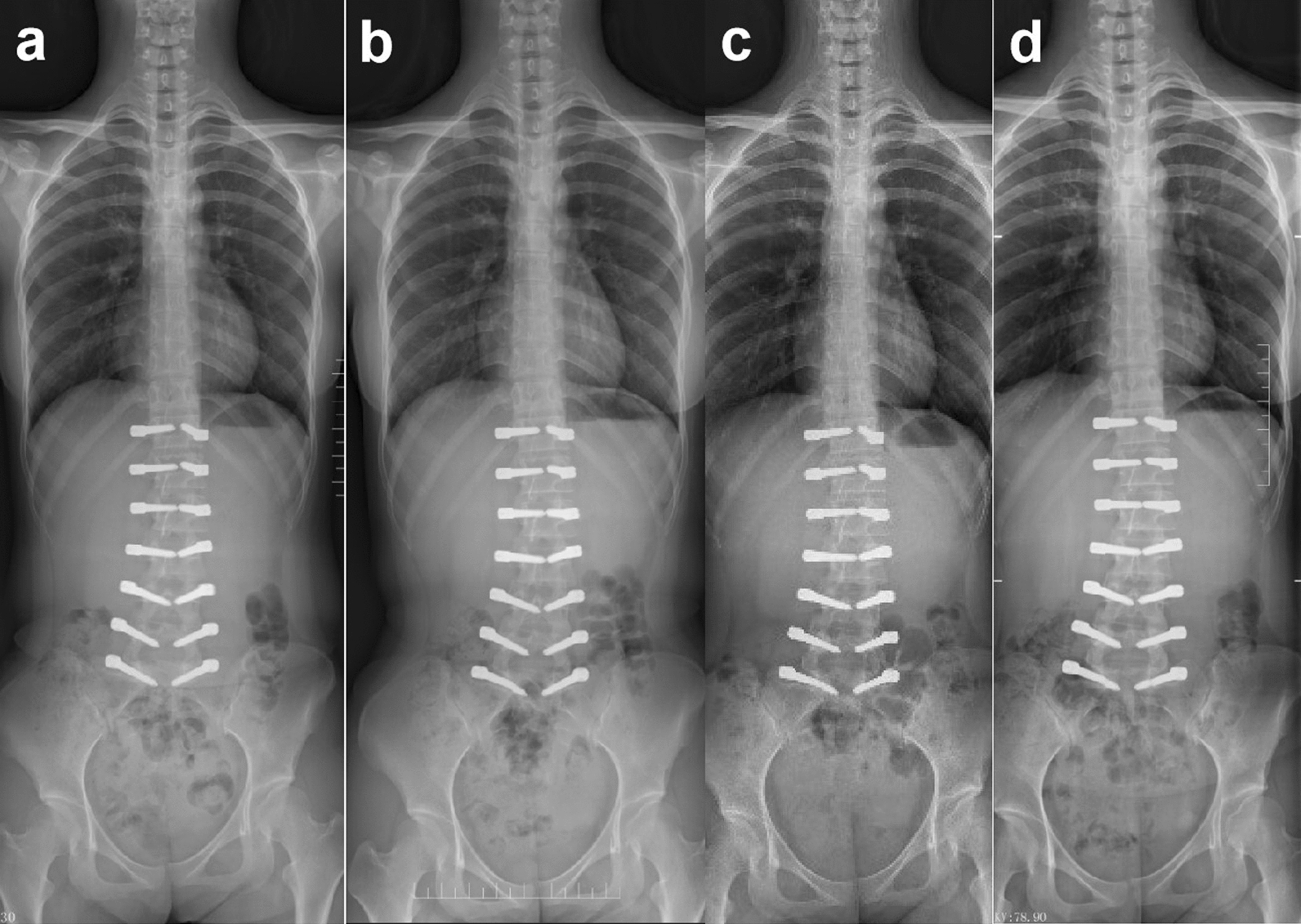
The halo sign appeared around the S1 screw 6 months postoperatively (**a**); at 1.5 years after the operation, the right screw at S1 was fractured (**b**); 3 years after the operation, both screws at S1 were fractured (**c**); 4 years after the operation, there was no significant progression of scoliosis (**d**)

**Fig. 6:**
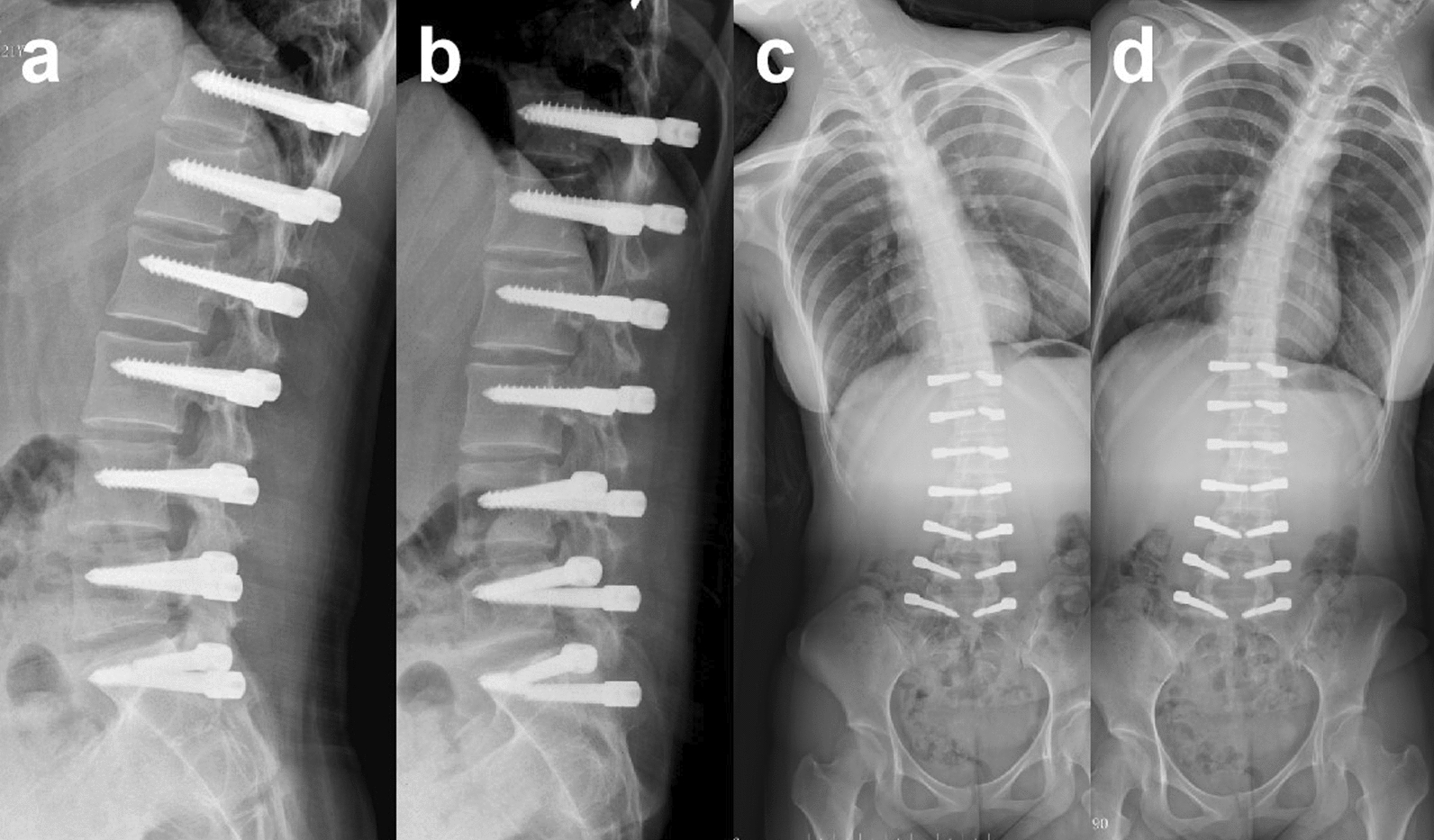
The sagittal range of motion was 15.3° (**a**, **b**), and the coronal range of motion was 7.6° (**c**, **d**) 4 years after the operation

**Fig. 7:**
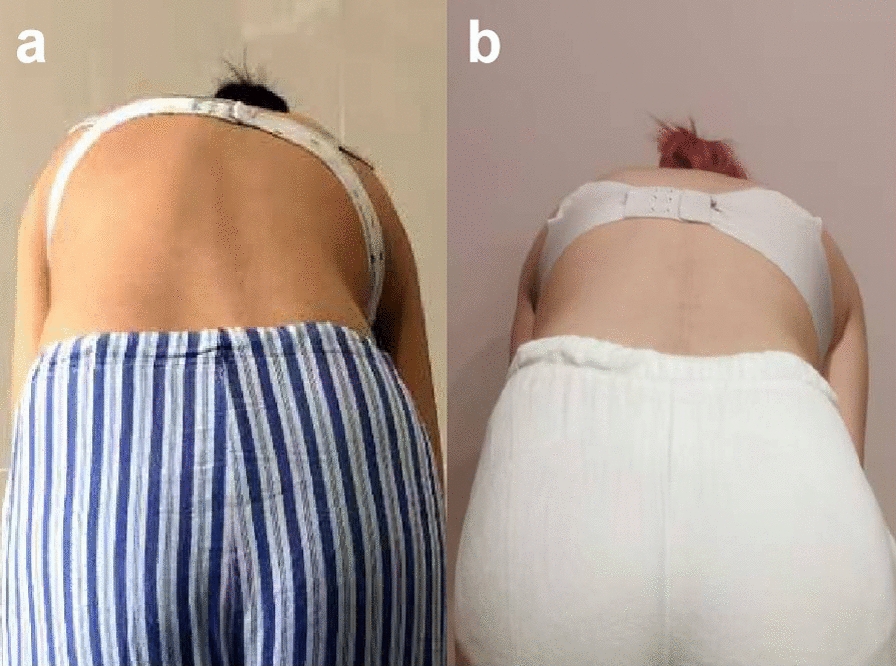
The prominence in the patient’s left lumbodorsal region was improved when bending forward; **a** pre-surgery; **b** over 5 years post-surgery

**Fig. 8:**
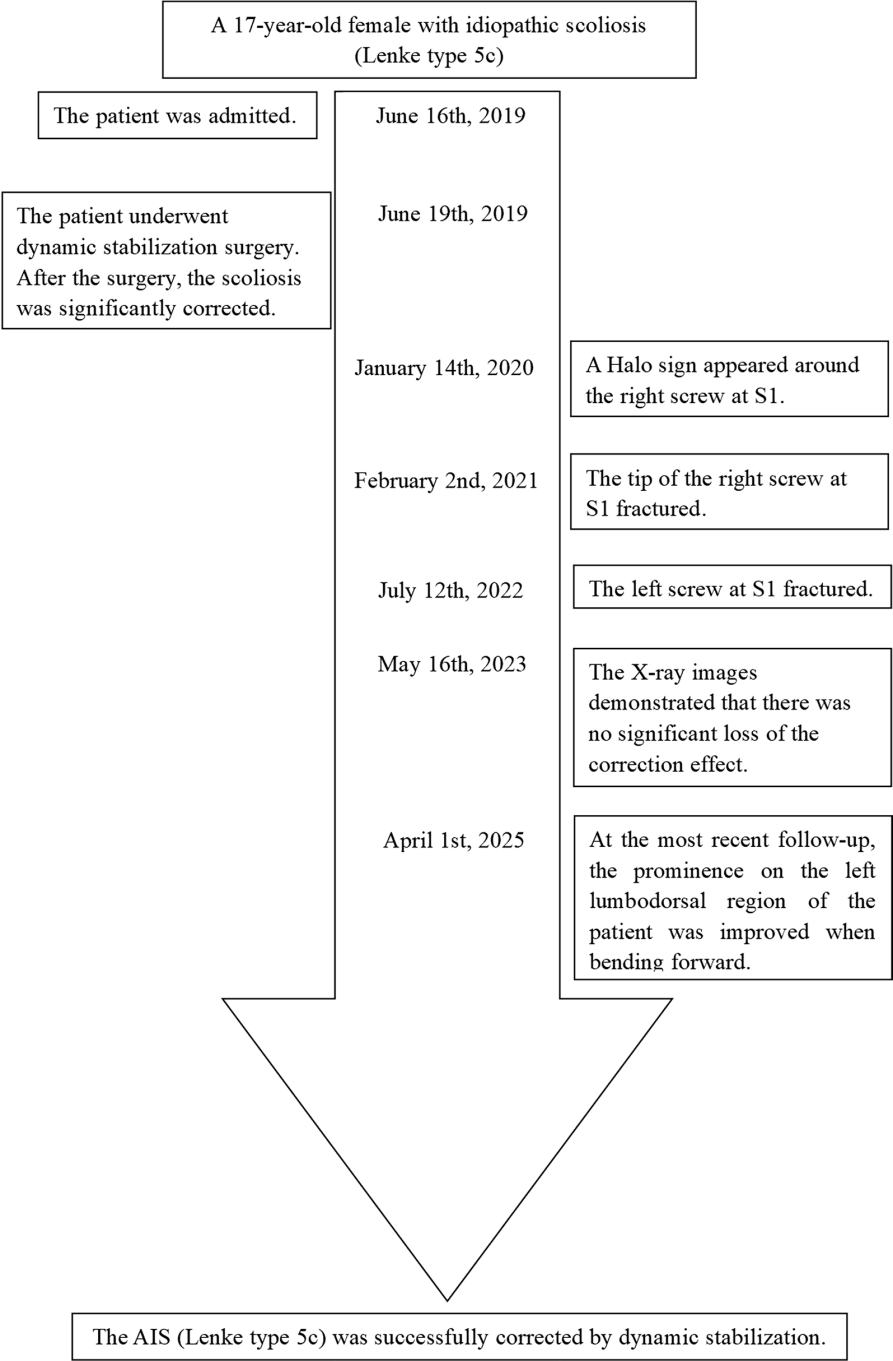
Case timeline

After the surgery, the low back pain was significantly relieved. We also achieved a satisfactory correction of the scoliosis. The Cobb angle of the lumbar major curve was 3.6° (correction rate 88.5%) and that of the lumbosacral curve was 0.4° postoperatively. The lumbar lordosis angle was 43.2° (Fig. 4). At 6 months after surgery, a halo sign appeared around the right screw at S1. At 1 year postoperatively, the tip of the right screw at S1 fractured. At 3 years after surgery, the left screw at S1 fractured. At 4 years after surgery, the Cobb angle of the lumbar curve was 6.1°, and the lumbosacral curve was 0.9°. Adequate correction was preserved (Fig. 5). The lumbar lordosis angle was 32.4°, the sagittal range of motion was 15.3°, and the coronal range of motion was 7.6° (Fig. 6). Although a Halo sign appeared around the bilateral S1 screws and they subsequently fractured, there was no obvious loss of correction. There were also no symptoms of low back pain, and the patient’s daily life and studies were not affected (Fig. 8). At the most recent follow-up (over 5 years postoperatively), the prominence in the patient’s left lumbodorsal region was improved when bending forward (Figs. 7b, 8).

## Discussion

At present, a preliminary consensus has gradually been reached regarding the strategies and techniques of instrumented fusion surgery for the treatment of AIS [13–15]. However, this currently widely used technology sacrifices the motion of the spine, especially for patients with Lenke type 5 AIS. In these patients, the main curvature is located in the thoracolumbar segments or lumbar segments, and the fusion range generally includes the upper and lower end vertebrae [16]. After surgery, patients’ activities of daily living, such as bending, squatting, wiping after defecation, among others, are likely to be affected [17]. In addition, owing to the loss of function in the fixed segments, the stress on adjacent segments increases, which makes them prone to degeneration [18]. Is instrumented fusion the only option in the surgical treatment of AIS? This patient, at the age of 17 years and with a Risser sign of grade 4, presented low potential for further skeletal growth. Consequently, bracing treatment has proven to be ineffective at this stage [19]. However, the scoliosis demonstrated relatively good flexibility, which was conducive to dynamic stabilization. Moreover, the development of soft tissues lags behind that of bone tissues by 1–2 years [20]. Hence, for this young patient, we attempted a nonfusion surgery. The results demonstrated that dynamic stabilization was capable of correcting the scoliosis, preventing further progression of the curve, and preserving motor function.

The determination to conduct surgical treatment for this patient was predicated upon a confluence of several factors. Firstly, this patient presented with recurrent low back pain and asymmetrical skin wrinkles in the lumbodorsal region, which had a negative impact on the patient’s appearance. At the age of 17 years, the appearance of the deformity often imposes psychosocial stress on the patient [21], which was also the patient’s primary concern. Secondly, the Cobb angle of scoliosis measured 32.2°, suggesting a potential influence on the lumbar intervertebral discs. The abnormal lumbar curvature may lead to asymmetrical loading and stress distribution in the intervertebral discs. This altered mechanical state has the propensity to accelerate disc degeneration over time. In addition, it was also noteworthy that the scoliosis might progress in middle and old age due to spinal degeneration [22]. Considering these factors, the patient opted for surgical treatment.

Dynamic stabilization surgery offers a series of advantages in the treatment of AIS that our case report confirmed. The Wiltse approach adopted in the surgical procedure allowed the operators to reach the screw entry point through the interspace between multifidus and longissimus without stripping the muscle enthesis. Compared with the traditional posterior midline approach, the Wiltse approach showed a lower incidence of multifidus atrophy and denervation, and less fatty infiltration [23]. Dynamic stabilization does not require facet joint osteotomy, removing the cortical bone on the surface of the laminae and posterolateral bone grafting; therefore, it has the advantages of short operation time and small intraoperative blood loss. Furthermore, in virtue of the absence of bone graft and the preservation of lumbar movement, a patient who undergoes dynamic stabilization is not required to wear a brace and is able to commence early mobilization, which is beneficial to the restoration of lumbar muscle function [24]. 

However, several drawbacks also exist. First of all, dynamic stabilization surgery is only applicable to patients with moderate scoliosis and very low spinal growth potential. Secondly, owing to the challenge of standardizing the length of the spacer and the tension of the cord, the surgery is relatively individualized. In addition, there is a risk of screw loosening and breakage. In this particular patient, the S1 screw fractured, yet the corrective effect was not lost during the postoperative follow-up.

## Conclusion

In the case, dynamic stabilization has been demonstrated to be technically feasible for the treatment of thoracolumbar/lumbar moderate scoliosis. The benefits are related to less damage to the soft tissues, reduced blood loss, and motion preservation. However, long-term outcomes are required before it can be recommended for routine use.

## Data Availability

Not applicable.
